# A Ternary Magnetic Recyclable ZnO/Fe_3_O_4_/g-C_3_N_4_ Composite Photocatalyst for Efficient Photodegradation of Monoazo Dye

**DOI:** 10.1186/s11671-019-2974-2

**Published:** 2019-04-29

**Authors:** Zhansheng Wu, Xiaoqing Chen, Xiaochen Liu, Xia Yang, Yan Yang

**Affiliations:** 1 School of Environmental and Chemical Engineering, Xi’an Polytechnic University, Xi’an, 710048 People’s Republic of China; 2 School of Chemistry and Chemical Engineering, Shihezi University, Shihezi, 832003 People’s Republic of China

**Keywords:** ZnO, g-C_3_N_4_, Photocatalytic degradation, Methyl orange

## Abstract

To develop a highly efficient visible light-induced and conveniently recyclable photocatalyst, in this study, a ternary magnetic ZnO/Fe_3_O_4_/g-C_3_N_4_ composite photocatalyst was synthesized for the photodegradation of Monas dye. The structure and optical performance of the composite photocatalyst were characterized using X-ray diffraction (XRD), transmission electron microscopye (TEM), energy dispersive spectroscopy (EDS), photoluminescence (PL) spectra, ultraviolet–visible diffuse reflection, and photo-electrochemistry. The photocatalytic activities of the prepared ZnO/Fe_3_O_4_/g-C_3_N_4_ nanocomposites were notably improved, and they were significantly higher than those of pure g-C_3_N_4_ and ZnO. Given the presence of the heterojunction between the interfaces of g-C_3_N_4_ and ZnO, the higher response to visible light and separation efficiency of the photo-induced electrons and holes enhanced the photocatalytic activities of the ZnO/Fe_3_O_4_/g-C_3_N_4_ nanocomposites. The stability experiment revealed that ZnO/Fe_3_O_4_/g-C_3_N_4_-50% demonstrates a relatively higher photocatalytic activity after 5 recycles. The degradation efficiency of MO, AYR, and OG over ZnO/Fe_3_O_4_/g-C_3_N_4_-50% were 97.87%, 98.05%, and 83.35%, respectively, which was due to the number of dye molecules adsorbed on the photocatalyst and the structure of the azo dye molecule. Azo dyes could be effectively and rapidly photodegraded by the obtained photocatalyst. Therefore, the environment-friendly photocatalyst could be widely applied to the treatment of dye contaminated wastewater.

## Introduction

As a major global environmental issue, a significant amount of pollutants is discharged into the lakes, rivers, and ground water due to rapid industrialization, which leads to water pollution. It was estimated that approximately 10–15% of organic dyes are discharged, which has carcinogenic and mutagenic effects on humans [1]. Therefore, methods that degrade industrial waste water, particularly organic dyes, are currently under investigation by researchers. Among various methods, the use of a photocatalytic technology with photocatalysts to degrade environmental pollutants was considered a potential approach [2, 3].

Furthermore, ZnO is one of the most widely used photocatalysis, given its high photosensitivity, low-cost, and environmentally friendly nature [4, 5]. However, pure ZnO is subject to three major drawbacks. First, it can only absorb ultraviolet (UV) light of solar energy with a wavelength less than 368 nm due to its wide band gap (3.37 eV), which limits its practical applications when sunlight is the energy source [6]. Second, a faster recombination of its photogenerated electron-hole pairs leads to a lower photocatalytic activity [7]. Third, the re-collection of ultrafine ZnO nanoparticles from the waste water using filtration and centrifugation is difficult to achieve, which limits its large-scale practical applications in the industry. Hence, in recent years, there were several attempts to develop multi-functional photocatalyts based on ZnO nanomaterials, with a high recyclability and excellent photocatalytic performances in the UV and visible irradiation ranges.

Different strategies were therefore implemented to overcome the first and second drawbacks of ZnO, such as doping, surface modification with metal nanoparticles, and the development of heterostructure [8–10]. Among these, coupling ZnO with a narrow band gap semiconductor with high conduction band (CB) can effectively increase the range of light absorption and accelerate the separation rate of the electron-hole pairs. Graphite-like carbon nitride (g-C_3_N_4_), which has a band gap of 2.70 eV, was explored as a promising metal-free material for the conversion of solar energy into electricity or chemical energy [11, 12]. Moreover, it attracted significant attention due to its excellent photocatalytic performance, chemical and thermal stabilities, and favorable electronic structure, given the strong covalent bonds between the carbon and nitrogen atoms. However, a high recombination rate of photo-induced electron-hole pairs limited its enhanced photocatalytic performance [13]. Wide-bandgap semiconductors could be combined with g-C_3_N_4_ to achieve improved charge separation [7, 14, 15]. Based the abovementioned methods, the combination of ZnO (wide-bandgap semiconductor) and g-C_3_N_4_ (narrow bandgap semiconductor) as a composite photocatalyst prevents the recombination of photogenerated electron–hole pairs and extends the light-absorption range of ZnO to the visible light spectrum. However, in most of the reported works, ZnO/g-C_3_N_4_ photocatalysts have low catalytic performance and are difficult to recovery and reusability. Fortunately, Fe_3_O_4_ was widely used in the preparation of magnetic photocatalysts, due to its good magnetic low-cost, good stability, and environment-friendly nature [16]. Hence, the preparation of novel visible-light-driven magnetic ZnO/Fe_3_O_4_/g-C_3_N_4_ photocatalysts is significant, and it is important to further improve the photocatalytic efficiency. In addition, how the structure of monoazo dyes affects the photodegradation process of the photocatalyst has not been reported yet. So it is very interesting to explore and provide a relaible theoretical basis for the application of photocatalysts in the efficient and fast treatment of dye wastewaters.

In this study, a novel and efficient photocatalyst of ZnO/Fe_3_O_4_/g-C_3_N_4_ nanocomposites was successfully prepared. The crystal structure, chemical states, and optical properties of the photocatalyst were characterized using X-ray diffraction (XRD), transmission electron microscopye (TEM), energy dispersive spectroscopy (EDS), X-ray photoelectron spectroscopy (XPS), photoluminescence (PL), vibrating sample magnetometry (VSM), and UV-vis diffuse reflectance spectroscopy (DRS). The photocatalytic performance of the photocatalyst was investigated by its degradation of methyl orange (MO) under visible light irradiation. The degradation of different monoazo dyes (MO, alizarin yellow R (AYR), and orange G (OG)) over ZnO/Fe_3_O_4_/g-C_3_N_4_ was also investigated. Moreover, to further evaluate the possible mechanism of the photocatalytic degradation of azo dyes, a free radical capture experiment and PL technique were employed.

## Materials and Methods

### Materials

Zinc acetate was supplied by Tianjin Fuchen Chemical Reagent Co., Ltd. (Tianjin, China); ethanol (EtOH) (anhydrous alcohol) was purchased from Tianjin Fuyu Fine Chemical Co., Ltd. (Tianjin, China); urea and oxalic acid were obtained from the Tianjin Shengao Chemical Industry Co., Ltd. (Tianjin, China); and MO, AYR, and OG were provided by Tianjin Yongsheng Fine Chemical Co., Ltd. (Tianjin, China). The selected properties of MO, AYR, and OG are presented in Table 1.

**Table 1:** Selected properties of azo dyes

Azo dyes	Structure	Formula	Molecular weight (g/mol)	Number of Sulfonic acid
AYR		C_13_H_8_N_3_NaO_5_	309.21	0
MO		C_14_H_14_N_3_SO_3_Na	327.33	1
OG		C_16_H_10_N_2_Na_2_O_7_S_2_	452.37	2

### Preparation of Fe_3_O_4_

For the reparation of Fe_3_O_4_, 0.540 g of FeCl_3_∙6H_2_O and 0.278 g of FeSO_4_∙7H_2_O (molar ratio 2:1) were dissolved in 40 mL of water. After 30 min of sonication, a brownish yellow solution was obtained and transferred to a 100-mL flask. Thereafter, the solution was stirred at 70 °C for 60 min in a nitrogen atmosphere, after which, 5 mL of aqueous ammonia (25%) was added to the solution under stirring. The obtained dark brown suspension was stirred for an additional 60 min and washed twice using water and ethanol, successively. The solid was then separated from the liquid phase using a magnetic field. The prepared dark brown sample was dried in an vacuum oven at 40 °C for 12 h.

### Preparation of ZnO/Fe_3_O_4_

The photocatalyst was prepared based previous studies [17]. In a representative synthesis, solution A was prepared using the method that involves the dissolution of zinc acetate (2.196 g) in ETOH (60 mL) and stirring at 60 °C in a water bath for 30 min. Moreover, solution B was obtained by adding 5.040 g of oxalic acid solution to 80 mL of ETOH under stirring at 50 °C for 30 min. Solution B was then added dropwise to the warm solution A and stirred continuously at room temperature for 1 h to obtain the sol. Thereafter, to obtain a homogenous gel, the sol was aged in a sealed environment for a period of time. The product was dried for 24 h in a vacuum oven at 80 °C. Finally, ZnO was obtained by thermal treatment at 400 °C for 2 h. To prepare ZnO/Fe_3_O_4_, 0.12 g of Fe_3_O_4_ was dispersed in solution A.

### Preparation of ZnO/Fe_3_O_4_/g-C_3_N_4_

For the preparation of ZnO/Fe_3_O_4_/g-C_3_N_4_, a homogeneous mixture was obtained by vigorously grinding 1 g of ZnO/Fe_3_O_4_ and melamine with a mass ratio of 1:1 and then dispersing the mixture in 20 ml of deionized water. The suspension was ultrasonicated for 1 h. Thereafter, the precursors were dried at 70 °C overnight to remove the solvent, and then the obtained solid was annealed at 550 °C for 2 h in air. The magnetic ZnO/Fe_3_O_4_/g-C_3_N_4_-50% composite was then successfully obtained. The amount of g-C_3_N_4_ was adjusted by controlling the amount of melamine (0.25 g, 1 g, and 2.3 g) during the preparation of the ZnO/Fe_3_O_4_/g-C_3_N_4_ nanocomposites, and the relevant products were denoted as ZnO/Fe_3_O_4_/g-C_3_N_4_-20%, ZnO/Fe_3_O_4_/g-C_3_N_4_-50%, and ZnO/Fe_3_O_4_/g-C_3_N_4_-70%, respectively.

### Characterization Methods

The XRD spectra of the samples were analyzed using an Rigaku Giegerflex D/Max B diffractometer with Cu-Kα radiation. The TEM was conducted together using a Tecnai G2F20 (USA) microscope. EDS spectra were performed by using an energy-dispersive X-ray spectrometer (EDS) attached to the TEM instrument. A surface area analyzer (Micromeritics, ASAP-2020, USA) was used to characterize the pore volume, pore size distribution, and specific surface area of the samples under N_2_ adsorption at 77 K. To determine the optical band gap of the photocatalys, the UV-visible absorption spectrum was obtained using a UV-Visible spectrophotometer with a reflectance standard of BaSO_4_ (Hitachi UV-4100, Japan). The surface composition and chemical states of the samples were investigated using XPS (250XI ESCA) equipped with an Mg Kα X-ray source (1253.6 eV). The PL spectra of the samples were determined using a fluorescence spectrophotometer (FLsp920, England) at room temperature, with an Xe lamp as an excitation light source. Photoelectrochemical measurements were conducted in three-electrode quartz cells with a 0.1-M Na_2_SO_4_ electrolyte solution. Platinum wire was used as the counter electrode, and Ag/AgCl were used as the reference electrodes, respectively. The working electrode was prepared as follows: 10 mg of the as-prepared photocatalyst was suspended in 1 mL of deionized water, which was then dip-coated onto a indium-tin oxide (ITO) glass electrode with dimensions of 10 mm × 20 mm and then dried under an infrared lamp.

### Photocatalytic Activity for Azo Dye

Photocatalytic experiments were conducted using a 500-W Xe lamp with a 420-nm cut-off filter at 25 °C, to study the visible light degradation of the MO, AYR, and OG solutions. In a traditional test, 10 mg of catalyst was added to 50 mL of azo dye solution (30 mg/L). The mixture was kept in the dark for 30 min to promote the adsorption of azo dye on the surface of the photocatalyst. The mixture was then irradiated under an Xe lamp to degrade the azo dye. After the degradation experiment, each sample was filtered with a 0.45 -μm filter membrane to remove the photocatalyst particles for analysis, and the concentrations of MO, AYR, and OG in the supernatant liquid were measured using a UV-5100 N spectrophotometer at *λ*max = 466 nm, 373 nm, and 475 nm, respectively. The degradation efficiency (*η*) of the azo dye was calculated as follows:

$$ \eta =\frac{C_0-{C}_{\mathrm{t}}}{C_0}\times 100\% $$

where C_0_ and C_t_ are the concentrations of the azo dye at the initial and specified irradiation times, respectively.

## Results and Discussion

### XRD

**Fig. 1:**
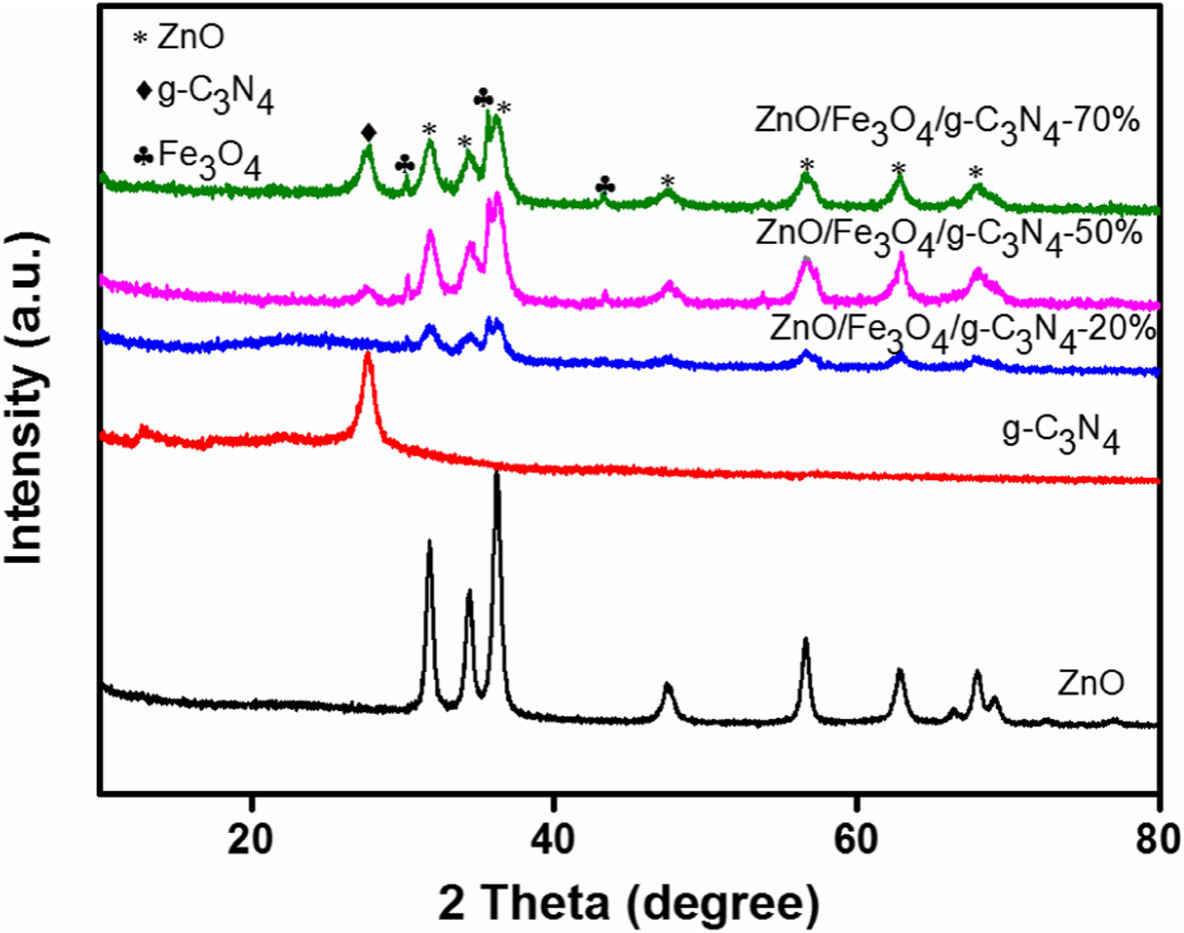
XRD patterns of ZnO, g-C_3_N_4_, ZnO/Fe_3_O_4_/g-C_3_N_4_-20%, ZnO/Fe_3_O_4_/g-C_3_N_4_-50%, and ZnO/Fe_3_O_4_/g-C_3_N_4_-70%

X-ray diffraction (XRD) analysis was used to study the phase structures of the ZnO, g-C_3_N_4_, and ZnO/Fe_3_O_4_/g-C_3_N_4_ composites with different g-C_3_N_4_ loadings. The results are presented in Fig. 1. The peaks of the ZnO samples located at 2*θ* = 31.81°, 34.44°, 36.21°, 47.60°, 56.62°, 63.01°, and 67.97° correspond to the (100), (002), (101), (102), (110), (103), and (112) crystal planes of the hexagonal wurtzite structure of ZnO. All relevant diffraction data for the ZnO were in good agreement with the JCPDS 36-145 [17]. The strongest peak of the g-C_3_N_4_ sample corresponds to the (002) plane of its layer structure at 2θ = 27.3°. As reported, the g-C_3_N_4_ structure has a weak diffraction peak at 2θ = 13.2°, which is attributed to the (100) crystal plane of g-C_3_N_4_. The width of the diffraction peak decreased, which indicates the influence of geometric constraints on the nanopore wall [7]. The XRD patterns of the ZnO/Fe_3_O_4_/g-C_3_N_4_-x samples included all the typical peaks of g-C_3_N_4_, ZnO, and Fe_3_O_4_. The diffraction peaks located at 30.4°, 35.7°, and 43.4° correspond to the (220), (311), and (400) planes of Fe_3_O_4_ [18, 19]. Moreover, the peak intensity of the characteristic peak of g-C_3_N_4_ was gradually strengthened with an increase in the amount of g-C_3_N_4_, whereas the peak intensity of ZnO and Fe_3_O_4_ gradually decreased. No g-C_3_N_4_ characteristic peak was observed in the ZnO/Fe_3_O_4_/g-C_3_N_4_-20% samples, which can be attributed to the low content of the g-C_3_N_4_ in the composite. From the XRD analysis results, no other peaks were observed in all the samples, thus confirming the high purity of the samples.

### TEM and EDS

**Fig. 2:**
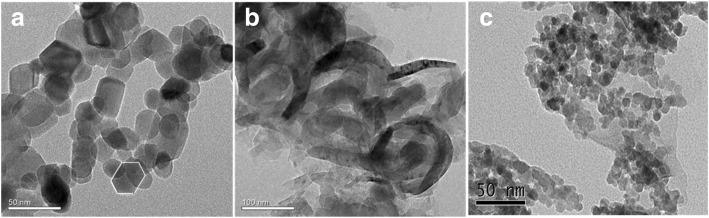
TEM images of **a** ZnO, **b** g-C_3_N_4_, and **c** ZnO/Fe_3_O_4_/g-C_3_N_4_-50%

**Fig. 3:**
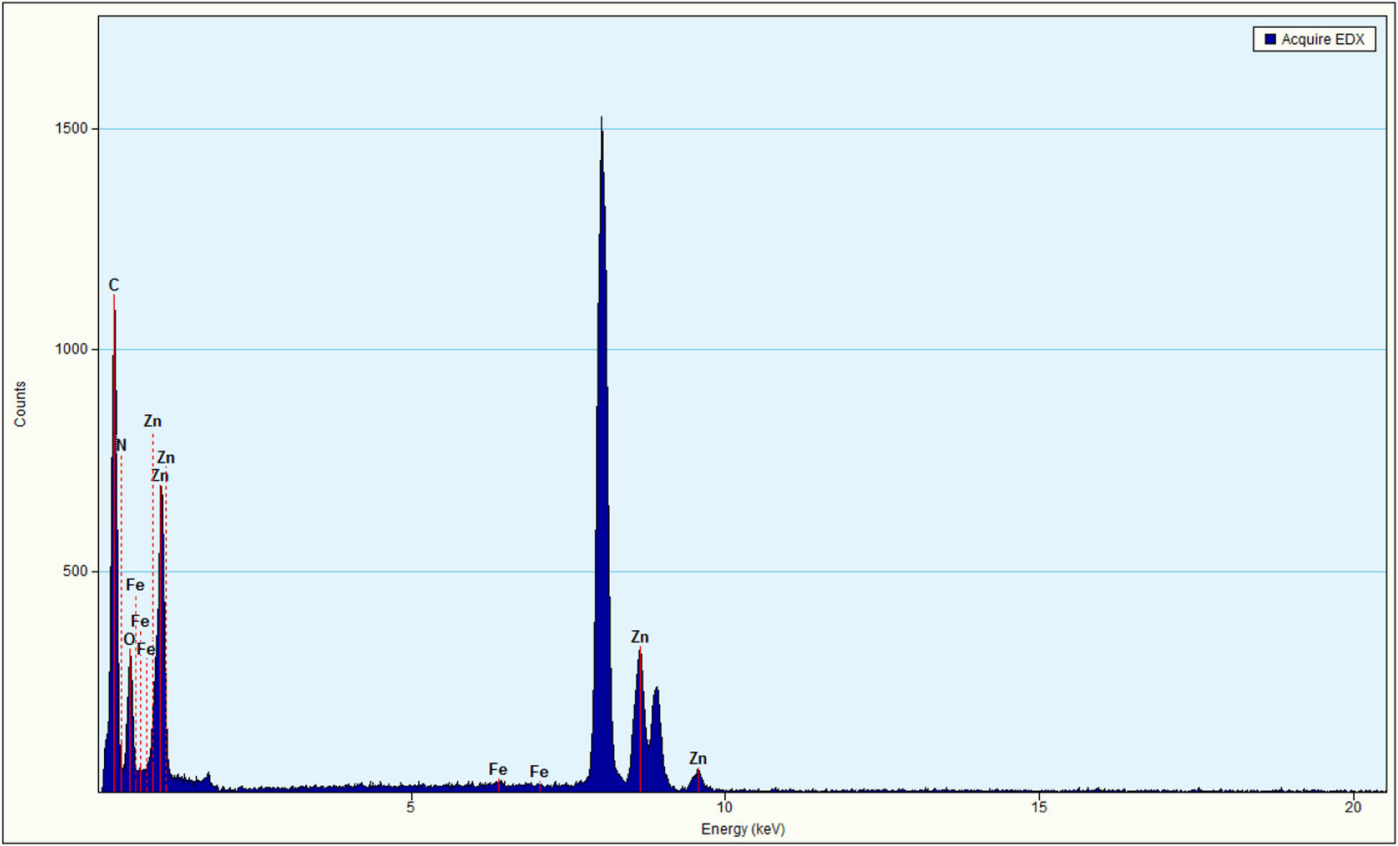
EDX of ZnO/Fe_3_O_4_/g-C_3_N_4_-50%

The structure of the sample was evaluated using TEM, as shown in Fig. 2. The TEM image of pure ZnO diplays the typical hexagonal wurtzite structure (Fig. 2a), which is consistent with the XRD results. The TEM image of g-C_3_N_4_ (Fig. 2b) displays its layered platelet-like morphology structure, and smooth paper-fold thinner sheets, which is similar to the morphology of graphene nanosheets. As seen from the TEM image of ZnO/Fe_3_O_4_/g-C_3_N_4_-50% (Fig. 2c), a large amount of photocatalysts accumulated on the layered structure of g-C_3_N_4_. The EDS results for ZnO/Fe_3_O_4_/g-C_3_N_4_-50% are presented in Fig. 3. It can be seen that the sample contained peaks of Zn, C, N, Fe, and O elements, which also proved that the ZnO/Fe_3_O_4_/g-C_3_N_4_ composite was prepared successfully. However, the peak value of Fe is relatively low, suggesting that the content of Fe_3_O_4_ is low in ZnO/Fe_3_O_4_/g-C_3_N_4_ composites. Given that Cu was used as a carrier in the TEM analysis, characteristic peaks of Cu were detected in the EDS analysis [20].

### XPS

**Fig. 4:**
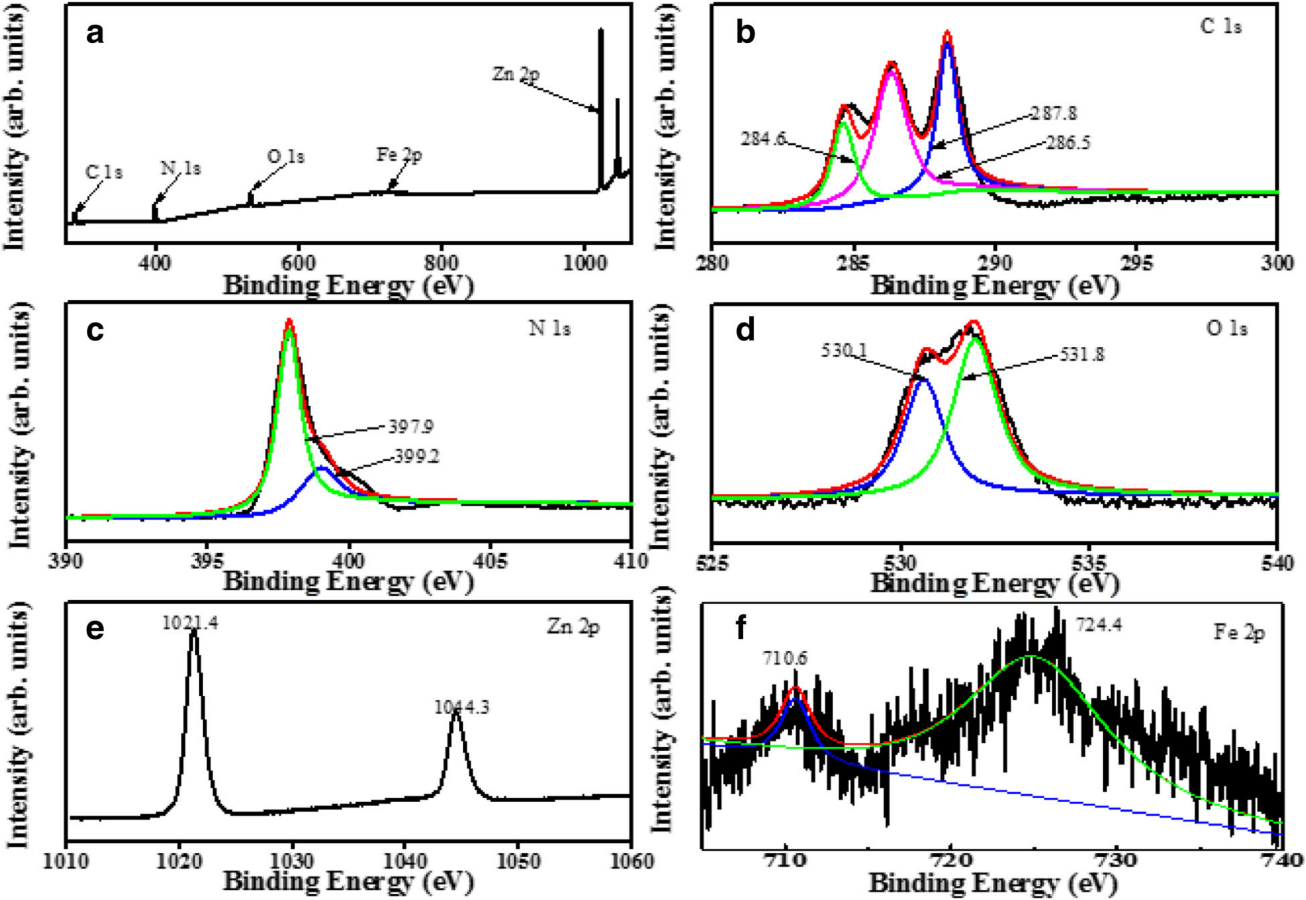
XPS spectra of the synthesized ZnO/Fe_3_O_4/_g-C_3_N_4_-50%: **a** survey of the sample, **b** C 1s, **c** N 1s, **d** O1s, **e** Zn 2p, and **f** Fe 2p

To investigate the surface composition and chemical states of the prepared composite catalysts, XPS was conducted on ZnO/Fe_3_O_4_/g-C_3_N_4_-50%. The survey spectrum scan reveals the presence of C, N, O, Zn, and Fe (Fig. 4a). Figure 4b reveals that the C 1s has three characteristic peaks. The peak located at 284.6 eV is attributed to the hydrocarbons in the XPS instrument and the sp2-hybridized carbon atoms in the aromatic ring, which were bonded to N (N–C=N). The other peak is attributed to the sp3 hybrid carbon source (C–(N)_3_) with a binding energy of 286.5 eV. The peak at the binding energy of 287.8 eV is attributed to the C–N–C in the graphite phase [21]. The N 1s XPS spectrum is presented in Fig. 4c. A major peak was at 397.9 eV, which correspond to the aromatic between N and two C atoms (C=N–C). A weaker characteristic peak is located at 399.2 eV, which is mainly attributed to the trinitrogen (N–(C)_3_) that links the basic structure (C_6_N_7_), or the amino groups related to the structural defects and incomplete condensation ( (C)_2_–N–H) [22]. The XPS spectrum of O 1s is presented in Fig. 4d, and the peak at 530.1 eV corresponds to the O_2_− ion in the Zn–O bond of the ZnO hexagonal wurtzite structure [23]. The peak at 531.8 eV corresponds to the oxygen vacancy in ZnO. In the Zn 2p XPS spectrum (Fig. 4e), there are two characteristic peaks at the binding energies of 1021.4 ev and 1044.3 eV, and the distance between the two peaks is 22.9 eV, which is included in the standard reference value of zinc oxide. The binding energy difference indicates that the zinc ion in the composite was in +2 states [23]. In the XPS spectrum of the Fe 2p (Fig. 4f), the two peaks are located at 710.6 ev and 724.4 eV, which correspond to the 2p1/2 and 2p3/2 orbitals, respectively [24]. These results reveal that g-C_3_N_4_ are composited on the ZnO, which may promote the absorption of visible light and improve the transfer and separation of charge carriers; thus, enhancing the photocatalytic activity [25].

### UV-vis DRS

**Fig. 5:**
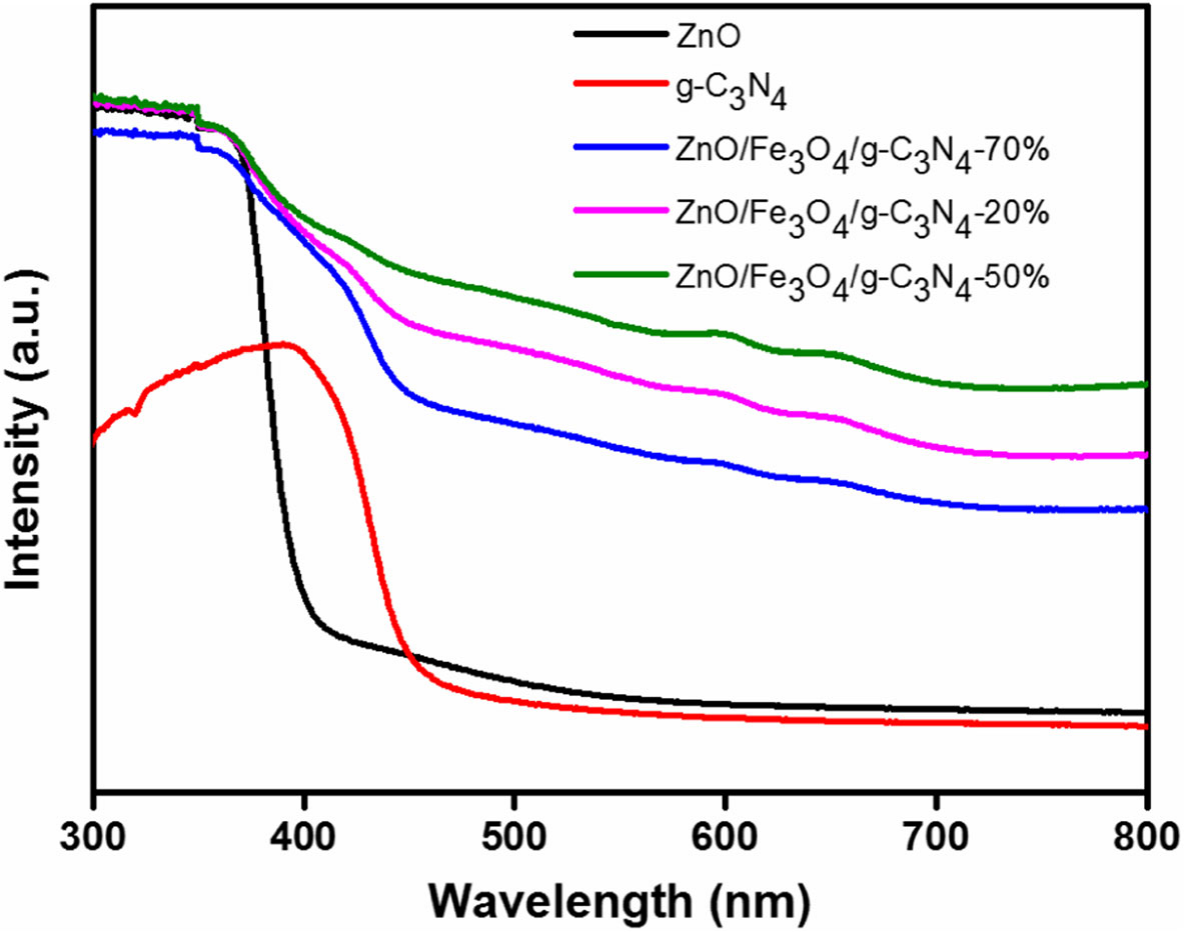
UV-vis diffuse reflectance spectra of ZnO, g-C_3_N_4_, ZnO/Fe_3_O_4_/g-C_3_N_4_-20%, ZnO/Fe_3_O_4_/g-C_3_N_4_-50%, and ZnO/Fe_3_O_4_/g-C_3_N_4_-70%

Diffuse reflectance spectroscopy was used to investigate the light absorption behavior of the photocatalysts. The results are presented in Fig. 5. The absorption of light with a significant red shift may improve photocatalytic performance in the visible region. In the ultraviolet region, the pure ZnO demonstrated a strong absorption at the wavelength of 388 nm, which corresponds to a band gap of 3.20 eV. Different from the ZnO absorption behavior, g-C_3_N_4_ yields an absorption shift at 460 nm, and the corresponding band gap energy was 2.70 eV, which indicates a higher response for photocatalytic activity under visible light [26]. Compared with pure ZnO or g-C_3_N_4_, the absorption edge of the ZnO/Fe_3_O_4_/g-C_3_N_4_ composite material shifted significantly to a longer wavelength region, which suggests that the absorption edge of the composite material shifted to the lower energy region. These results may be due to the synergistic relationship between g-C_3_N_4_ and ZnO in the composite samples, which is consistent with the report by Le et al. [7]. The red shift of the absorption edge of ZnO/Fe_3_O_4_/g-C_3_N_4_ increased with an increase in the g-C_3_N_4_ loading up to 50%. However, the absorption edge decreased, when the g-C_3_N_4_ loading was 70%. The decrease in ZnO/Fe_3_O_4_/g-C_3_N_4_-70% may be because g-C_3_N_4_ loading above the optimal level may shield the light intensity absorption by ZnO. Therefore, among all the prepared samples, the ZnO/Fe_3_O_4_/g-C_3_N_4_-50% composite exhibited the most extensive and strongest absorption of visible light. This is similar to the results obtained by Jo et al., who reported that ZnO–50%/g-C_3_N_4_ exhibited the strongest absorption of visible light [1]. The composite material demonstrated the strongest light absorption to visible light, which increased the generation of electron-hole pairs under visible light irradiation, resulting in a higher photocatalytic activity.

### PL

**Fig. 6:**
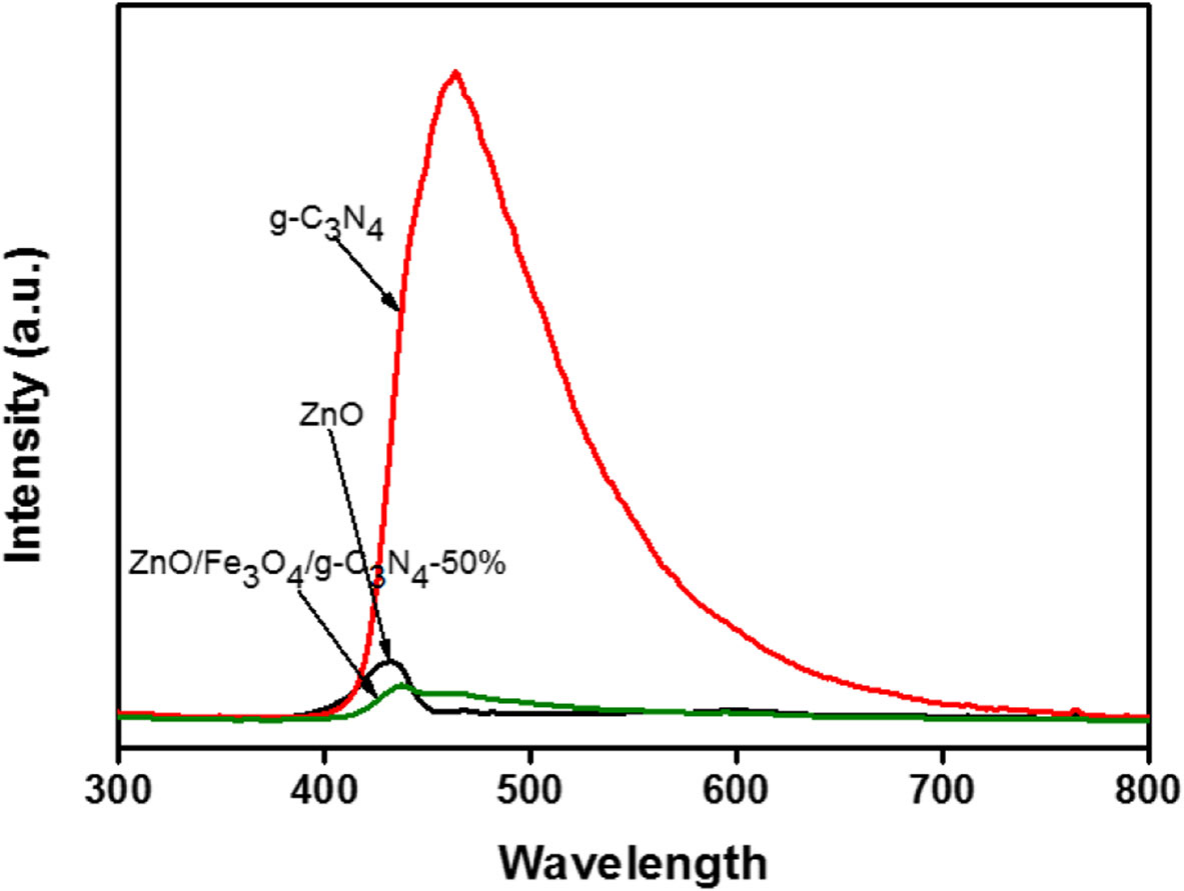
PL spectra of ZnO, g-C_3_N_4_, and ZnO/Fe_3_O_4_/g-C_3_N_4_-50%

The effect of the synergistic relationship between ZnO and g-C_3_N_4_ on the photocatalysis was further evaluated using PL. The PL spectra of ZnO, g-C_3_N_4,_ and ZnO/Fe_3_O_4_/g-C_3_N_4_-50% are presented in Fig. 6. The excitation wavelength was 300 nm, and the PL of the samples were tested at room temperature. The emission spectra in the range of 300–800 nm were recorded. It is common knowledge that the recombination of electron-hole pairs inside semiconductors releases energy in the form of PL. In general, a lower PL intensity indicates a lower recombination rate of carriers, which leads to efficient photocatalytic activity. In the PL spectrum, g-C_3_N_4_ exhibited a strong emission peak at approximately 460 nm, which is in accordance with the UV-vis results (Fig. 5) and literature [7]. The emission peak of pure ZnO was lower than that of g-C_3_N_4_, at approximately 410 nm [21]. Compared with the PL peak of pure ZnO, the emission peak of the ZnO/Fe_3_O_4_/g-C_3_N_4_-50% composite photocatalyst was red-shifted, and its peak intensity was significantly reduced. Moreover, PL peak intensity of the ZnO/Fe_3_O_4_/g-C_3_N_4_-50% composite photocatalyst was lowest. Based on these results, it was concluded that the electron-hole pairs photogenerated by the ZnO/Fe_3_O_4_/g-C_3_N_4_-50% nanocomposites under visible light irradiation can be effectively transferred at the interface of the heterostructure. Thus, the electron-hole recombination rate decreased, which resulted in the highest photocatalytic activity under visible light irradiation.

### Electrochemical Analysis

**Fig. 7:**
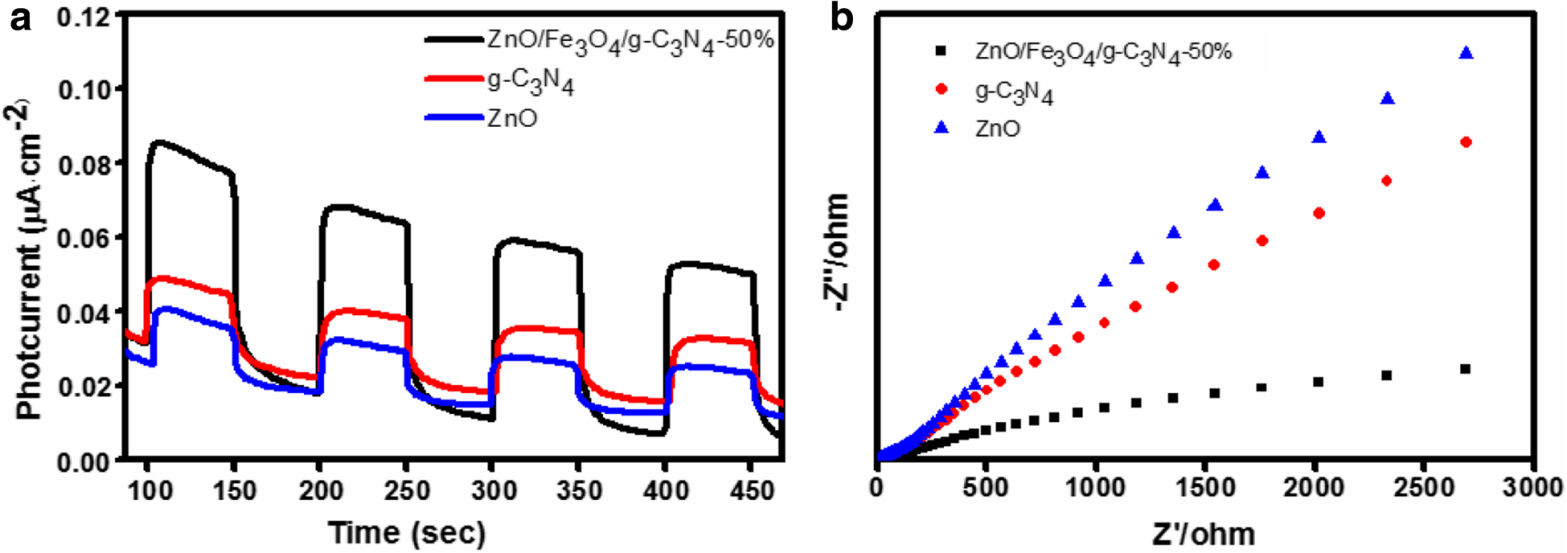
Transient photocurrent response (**a**) and EIS plots (**b**) ZnO, g-C_3_N_4,_ and ZnO/Fe_3_O_4_/g-C_3_N_4_-50% under visible-light irradiation

The photocatalytic redox reactions between the separation, migration, and capture of photogenerated electrons by semiconductor photocatalysts are closely related. To qualitatively evaluate the photo-induced charge separation efficiency during the photocatalytic reaction, the photocurrent responses of the ZnO, g-C_3_N_4,_ and ZnO/Fe_3_O_4_/g-C_3_N_4_-50% nanocomposites were investigated under visible light irradiation. Figure 7a presents the photocurrent-time (I-t) curves of three samples under intermittent illumination. From the figure, it can be seen that once the irradiation of light was turned off, the photocurrent value decreased abruptly, and the photocurrent maintained a constant value when the light was turned on again. Moreover, this phenomenon is reproducible, which indicates that most of the photogenerated electrons were transferred to the surface of the sample, and a photocurrent was generated under visible light irradiation. Pure ZnO demonstrates the weakest photocurrent response under visible light irradiation, due to its wide band gap. Moreover, the ZnO/Fe_3_O_4_/g-C_3_N_4_-50% composite samples exhibited the highest photocurrent intensities. The results suggest that the relationship between ZnO and g-C_3_N_4_ is beneficial for the improvement of the separation efficiency and transfer of photogenerated electrons and holes [27]. This phenomenon is consistent with the PL results.

The electrochemical impedance spectroscopy (EIS) results of the sample are presented in Fig. 7b. The arcs on the EIS electrochemical impedance spectrogram reflect the charge transfer layer resistance at the electrode/electrolyte interface. A smaller arc represents a lower resistance, which indicates a higher efficiency of charge transfer [27]. The arc radius of the ZnO/Fe_3_O_4_/g-C_3_N_4_-50% composite photocatalyst is smaller than that of ZnO and g-C_3_N_4_, which indicates that the charge transfer layer resistance of the ZnO/Fe_3_O_4_/g-C_3_N_4_-50% interface was the smallest. Thus, the photo-induced electron-hole pairs exhibited the highest separation and transfer efficiency, which improved the photocatalytic activity. These results are consistent with the photocurrent results.

### Magnetic Aroperties

**Fig. 8:**
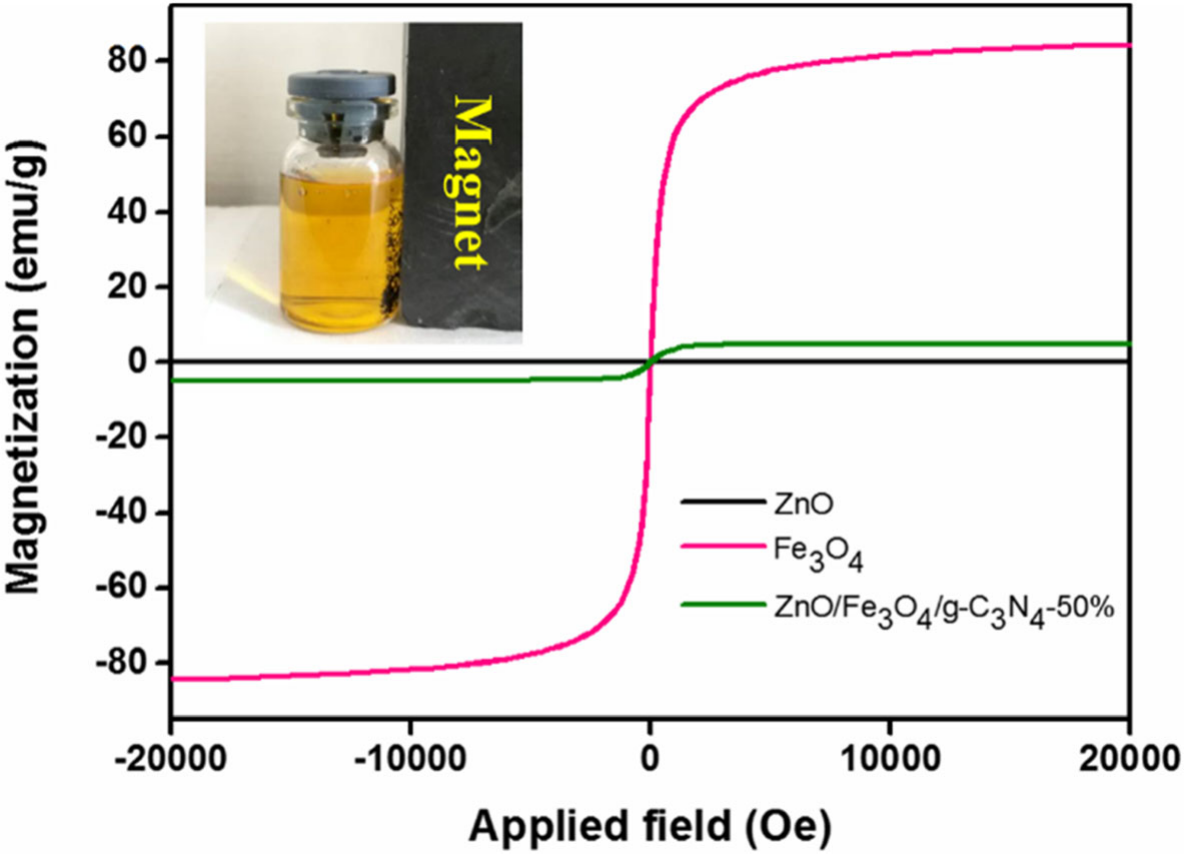
Magnetic hysteresis curves of ZnO, and ZnO/Fe_3_O_4_/g-C_3_N_4_-50% spectra at room temperature

The hysteresis loops of ZnO, Fe_3_O_4_, and ZnO/Fe_3_O_4_/g-C_3_N_4_-50% are presented in Fig. 8. The results reveal that pure ZnO is non-magnetic, pure Fe_3_O_4_ exhibited the strongest saturation magnetization, and the saturation magnetization of ZnO/Fe_3_O_4_/g-C_3_N_4_-50% was lower than that of the pure Fe_3_O_4_, which is attributed to the presence of non-magnetic substances, i.e., ZnO and g-C_3_N_4_. No hysteresis, remanence, and coercivity were observed in the hysteresis loop of ZnO/Fe_3_O_4_/g-C_3_N_4_-50%. Therefore, the sample was superparamagnetic. Moreover, the saturation magnetization of the composite photocatalyst was sufficient to separate from the solution using an external magnetic field, as shown in Fig. 8 (inset), which promoted the photocatalyst recovery and increased its recyclability.

### Photocatalytic Properties

**Fig. 9:**
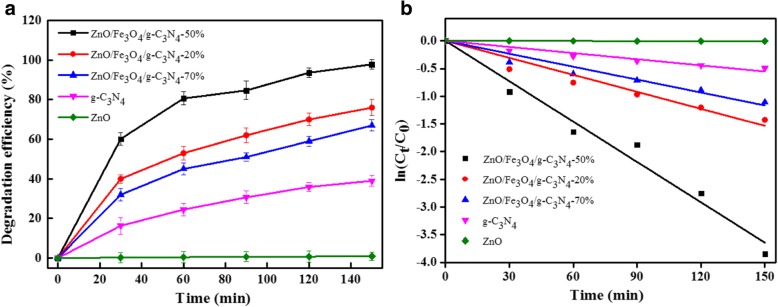
Photocatalytic degradation efficiency (**a**) and kinetic curves (**b**) of MO over different photocatalysts under visible light irradiation, (the relative error of the data was less than 5%)

The degradation of MO over different photocatalysts is presented in Fig. 9a. The pure ZnO slightly degraded the methyl orange under visible light irradiation, given that the wide band gap of ZnO allows it to respond only to ultraviolet light. The degradation efficiency of pure g-C_3_N_4_ for methyl orange was not very high, due to its high photoelectron-hole pair recombination rate, despite its response to visible light, which resulted in the low photocatalytic activity of g-C_3_N_4_. The photodegradation efficiency of MO on the ZnO/Fe_3_O_4_/g-C_3_N_4_-50% composite photocatalyst was higher than that of the other catalysts, for the following three reasons: First, the UV-Vis spectra indicated that the ZnO/Fe_3_O_4_/g-C_3_N_4_-50% composite photocatalyst exhibited the strongest visible light response intensity and a large visible light absorption range. Second, the PL and electrochemical results revealed that the electron-hole pair recombination rate of ZnO/Fe_3_O_4_/g-C_3_N_4_-50% was the lowest. Third, the electrochemical results indicated that the photoelectron transfer rate of theZnO/Fe_3_O_4_/g-C_3_N_4_-50% photocatalyst was the fastest compared with single photocatalyst.

In addition, the kinetics of the degradation of MO on the photocatalysts were also evaluated (Fig. 9b). The results revealed that the degradation kinetics of MO on different photocatalysts followed the first-order kinetic model, and all the degradation rate constants are presented in Table 2. The apparent rate constant of ZnO/Fe_3_O_4_/g-C_3_N_4_-50% was the highest (0.02430 min^−1^), and which was higher than the degradation rate of g-C_3_N_4_/Fe_3_O_4_/TiO_2_ and TiO_2_/biochar composite catalysts [28, 29]. Moreover, the ZnO/Fe_3_O_4_/g-C_3_N_4_-50% exhibited a higher photocatalytic rate relative to g-C_3_N_4_/Fe_3_O_4_/AgI on the degradation of MO (0. 0016 min^−1^) [10].

**Table 2:** First order kinetic constants and relative coefficients for photocatalytic degradation of MO over the samples

Samples	*k* (min^−1^)	*R* ^2^
ZnO	0.00006	0.9966
g-C_3_N_4_	0.00369	0.9779
ZnO/Fe_3_O_4_/g-C_3_N_4_-20%	0.01023	0.9827
ZnO/Fe_3_O_4_/g-C_3_N_4_-50%	0.02430	0.9904
ZnO/Fe_3_O_4_/g-C_3_N_4_-70%	0.00778	0.9819

### Stability of ZnO/Fe_3_O_4_/g-C_3_N_4_-50% Composite Photocatalyst

**Fig. 10:**
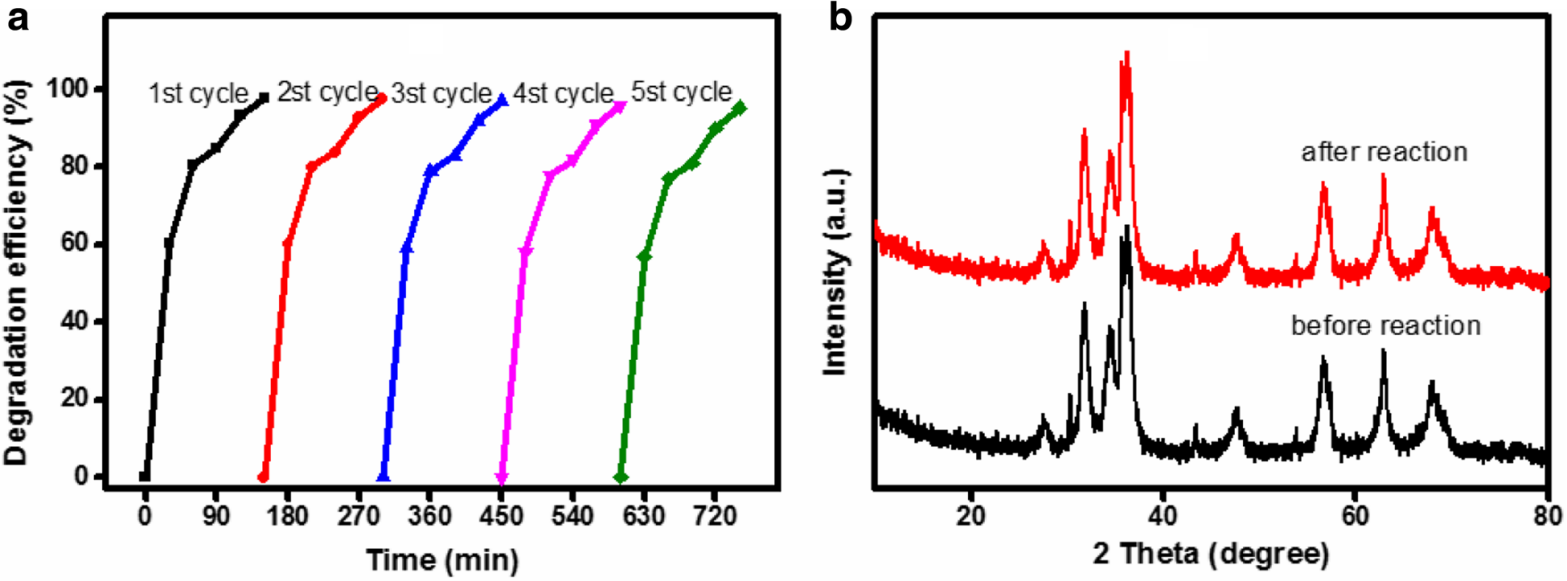
The recycling degradation efficiency (**a**) and XRD patterns of before and after degradation (**b**) of ZnO/Fe_3_O_4_/g-C_3_N_4_-50% for MO under visible light irradiation

In addition, the stability of photocatalysts is a critical factor in relation to large-scale technology application. To evaluate the stability of the ZnO/Fe_3_O_4_/g-C_3_N_4_-50% composite photocatalyst, recycling experiments were conducted on the photocatalyst for the degradation of MO under visible light irradiation. The photocatalyst was collected by magnetic decantation and then washed using distilled water and ethanol. Thereafter, it was dried in an oven at 80 °C. The sample was reused for subsequent degradation, and the results are presented in Fig. 10a. The composite photocatalyst maintained a very high photocatalytic activity, and the removal rate of MO on the ZnO/Fe_3_O_4_/g-C_3_N_4_-50% composite photocatalyst was 95.3% after 5 cycles. In addition, there was a slight decrease in the amount of photocatalysts during the cycle processes. Therefore, the ZnO/Fe_3_O_4_/g-C_3_N_4_-50% composite photocatalyst exhibited high stability under visible light irradiation. To further evaluate the stability of the ZnO/Fe_3_O_4_/g-C_3_N_4_-50%, samples were collected after 5 cycles for XRD testing and compared with the XRD pattern of the sample before cycling. The results are presented in Fig. 10b. No significant changes were observed in the structure of the photocatalyst before and after use, which indicates that the ZnO/Fe_3_O_4_/g-C_3_N_4_-50% photocatalyst was highly stable.

### Degradation of Monoazo Dyes on ZnO/Fe_3_O_4_/g-C_3_N_4_-50%

**Fig. 11:**
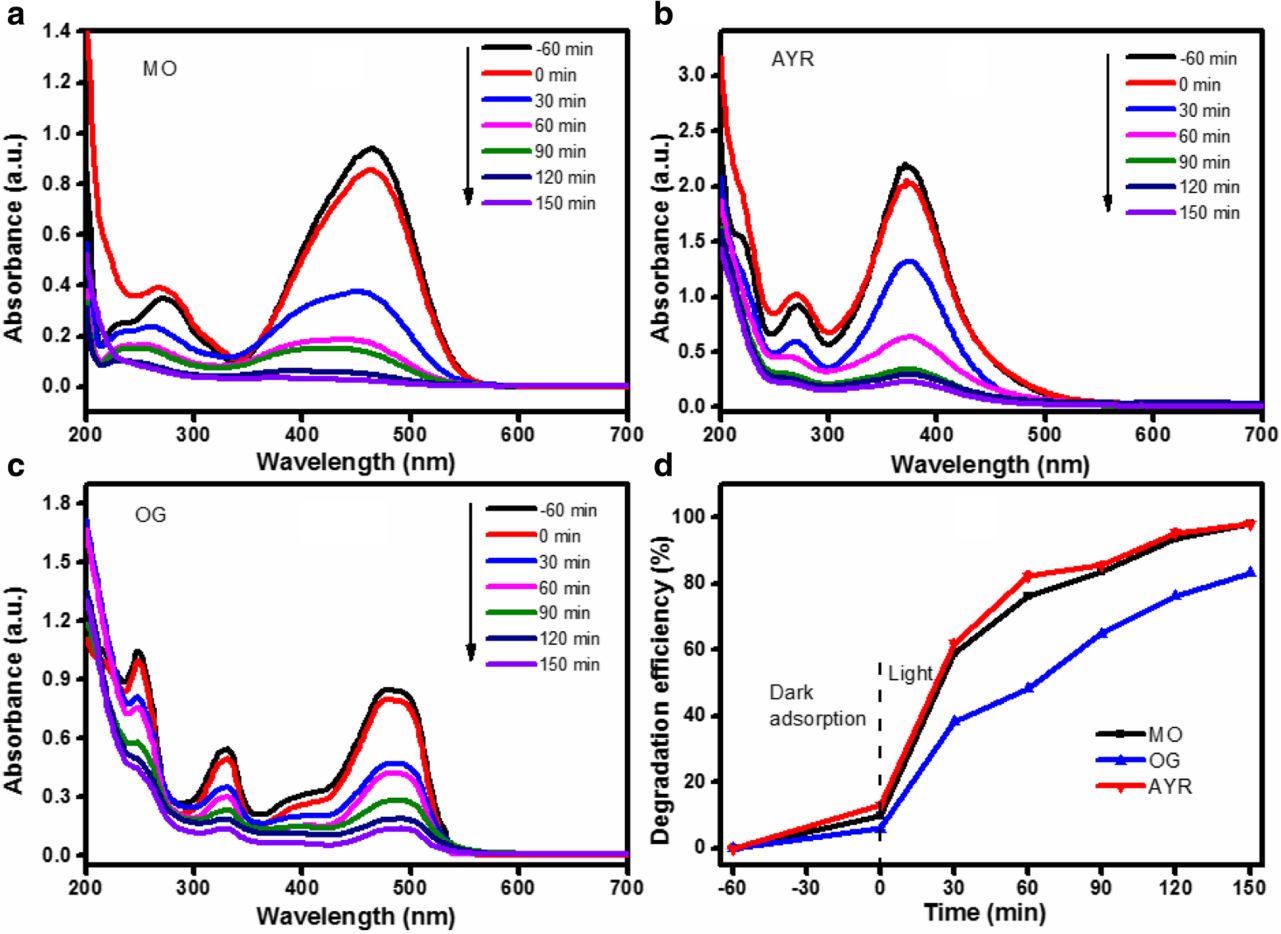
UV-vis spectra of **a** MO, **b** AYR, and **c** OG at different irradiation times, and **d** degradation efficiency curves of different dyes in the presence of ZnO/Fe_3_O_4_/g-C_3_N_4_-50%

For the evaluation of the photocatalytic degradation behavior of different monoazo dyes, the degradation of MO, AYR, and OG over ZnO/Fe_3_O_4_/g-C_3_N_4_ is presented in Fig. 11. The plots of the absorbance with respect to the wavelength for the MO, AYR, and OG degradations over ZnO/Fe_3_O_4_/g-C_3_N_4_-50% at various irradiation times are presented in Figs. 11a–c. The maximum absorption wavelength of MO, AYR, and OG before and after degradation were 466 nm, 372 nm, and 475 nm, respectively. With the gradual extension of the illumination time to 150 min, the intensity of the absorption peak gradually decreased, implying the gradual mineralization of MO, AYR, and OG. Furthermore, under visible-light irradiation for 150 min, the degradation efficiencies of MO, AYR, and OG were 97.87%, 98.05%, and 83.35%, respectively (Fig. 11d). There are two possible reasons for this phenomenon. First, as can be seen in Fig. 11d, the adsorption efficiency of OG on the photocatalyst was the lowest. The lower adsorption efficiency of OG can be explained by the steric limit of a large aromatic molecule, which reduced the number of OG molecules adsorbed on the photocatalyst. The lower adsorption efficiency of the azo dye therefore resulted in a small amount of molecules concentrated on the active site of the photocatalyst, which decreased the degradation efficiency of the azo dye [30]. Second, AYR has a high degradation efficiency, which is related to the presence of a carboxyl group that can react with H^+^ in a light Kolbe reaction. However, the lower degradation efficiency of OG and MO could be due to the presence of a withdrawing SO_3_^−^ group, and the increasing number of sulfonic acid groups could inhibit degradation of the dye [31]. The properties of the three dyes are listed in Table 1. The molecular weight and number of sulfonic acid was in the following order: AYR < MO < OG. Therefore, the degradation efficiency of OG over ZnO/Fe_3_O_4_/g-C_3_N_4_-50% was the lowest.

**Fig. 12:**
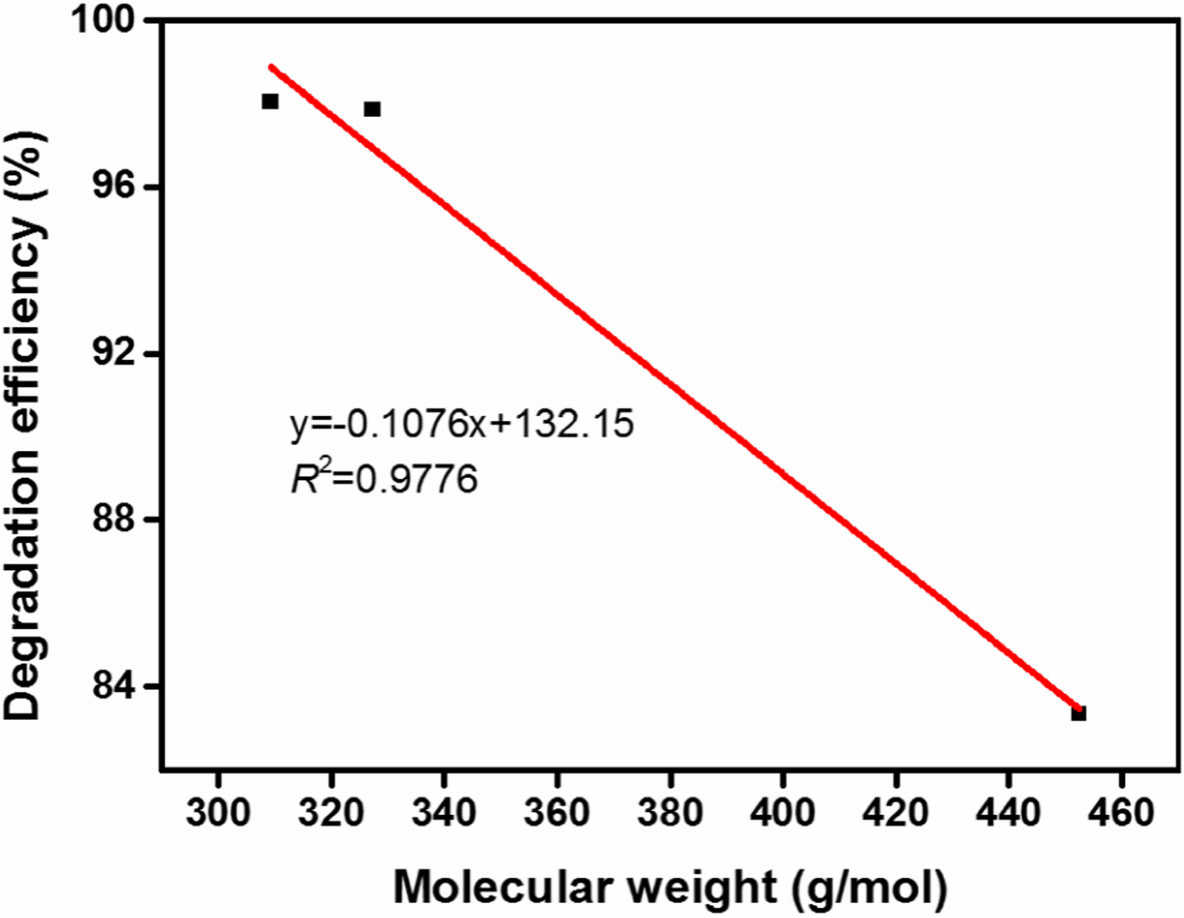
Correlation between molecular weight of azo dye and degradation efficiency

It is necessary to investigate the relationship between the molecular weight of the azo dye and its degradation efficiency. Figure 12 reveals that the molecular weight of azo dye had a good negative correlation with the degradation efficiency (*R*^2^ = 0.9776). Moreover, a molecular weight of the azo dye would result in a low degradation efficiency. The results are consistent with those presented above.

### Mechanism for Photocatalytic Degradation

**Fig. 13:**
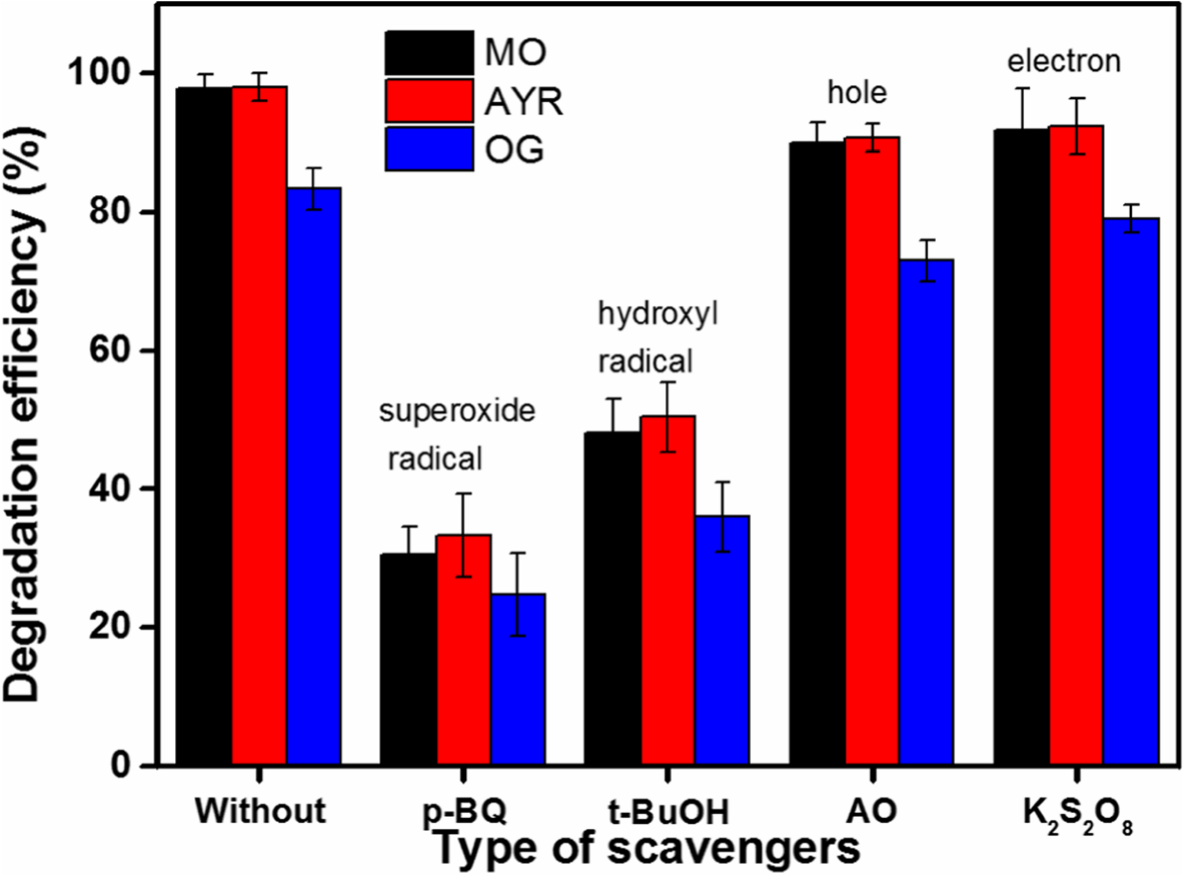
The degradation efficiencies of monazo dyes over ZnO/Fe_3_O_4_/g-C_3_N_4_-50% in the presence of various scavengers

To further investigate the mechanism of the photocatalyst for the degradation of MO under visible light irradiation, radical, electron, and hole scavenging experiments were conducted to detect the main active species in the photocatalytic process. Moreover, ·OH, ·O_2_^−^, h^+^, and e^−^ were eliminated using tert-butanol (*t*-BuOH), p-benzoquinone (*p*-BQ), ammonium oxalate (AO), and K_2_S_2_O_8_, respectively. The degradation efficiencies of MO on the photocatalyst in the presence the scavengers are presented in Fig. 13. The removal rate of MO was significantly reduced after the addition of *t*-BuOH and *p*-BQ. Conversely, the removal efficiency of MO was not significantly reduced in the presence of AO and K_2_S_2_O_8_. Therefore, the active species that play a critical roles during the photocatalytic degradation of MO over the ZnO/Fe_3_O_4_/g-C_3_N_4_-50% photocatalyst are ·OH and ·O_2_^−^

Based on the relevant literature and experimental results (including the physicochemical properties, photocatalytic performance, and detected active components), a possible photocatalytic mechanism of the ZnO/Fe_3_O_4_/g-C_3_N_4_-50% nanocomposites prepared under visible light irradiation is proposed. It is common knowledge that ZnO and g-C_3_N_4_ are typical n-type semiconductors. Therefore, an n–n heterojunction is formed at the interface between the g-C_3_N_4_ and ZnO nanoparticles. The ZnO/Fe_3_O_4_/g-C_3_N_4_-50% can be excited to generate electrons and holes under visible light irradiation. The excited electrons are then transferred from the CB of the g-C_3_N_4_ to the CB of the ZnO. The improvement of the photocatalytic performance of the composite photocatalyst is mainly due to the effective separation of photogenerated electrons and holes at the heterojunction interface [32]. Given that the CB edge potential of g-C_3_N_4_ is more negative than that of ZnO, the excited electrons in g-C_3_N_4_ are transferred to the CB of ZnO, and the holes are retained in the valence band (VB) of g-C_3_N_4_ [33, 34]. In contrast, ZnO holes are injected into the holes of g-C_3_N_4_. Therefore, an internal electrostatic potential is formed in the space charge region, which promotes the separation of the photogenerated carriers. The charge transfer to the surface of the compound semiconductor reacts with water and dissolved oxygen to produce ·OH and ·O_2_^−^, or it reacts directly with MO. From Fig. 13, it can be seen that ·OH and ·O_2_^−^ play a vital role in the degradation of MO on composite photocatalysts. Therefore, possible photocatalytic mechanisms are presented below:

$$ {\displaystyle \begin{array}{l}\mathrm{ZnO}/{\mathrm{Fe}}_3{\mathrm{O}}_4/\mathrm{g}\hbox{-} {\mathrm{C}}_3{\mathrm{N}}_4\hbox{-} 50\%+ h\nu \to {\mathrm{e}}_{\mathrm{C}\mathrm{B}}^{-}\left(\mathrm{ZnO}\right)+{\mathrm{h}}_{\mathrm{VB}}^{+}\left(\mathrm{g}\hbox{-} {\mathrm{C}}_3{\mathrm{N}}_4\right)\\ {}{\mathrm{e}}_{\mathrm{C}\mathrm{B}}^{-}+{\mathrm{O}}_2\to \cdot {\mathrm{O}}_2^{-}\\ {}{\mathrm{h}}_{\mathrm{VB}}^{+}+{\mathrm{H}}_2\mathrm{O}\to \cdot \mathrm{OH}+{\mathrm{H}}^{+}\\ {}\cdot \mathrm{OH}+\mathrm{MO}\to {\mathrm{H}}_2\mathrm{O}+{\mathrm{C}\mathrm{O}}_2\\ {}\cdot {\mathrm{O}}_2^{-}+\mathrm{MO}\to {\mathrm{H}}_2\mathrm{O}+{\mathrm{C}\mathrm{O}}_2\end{array}} $$

**Fig. 14:**
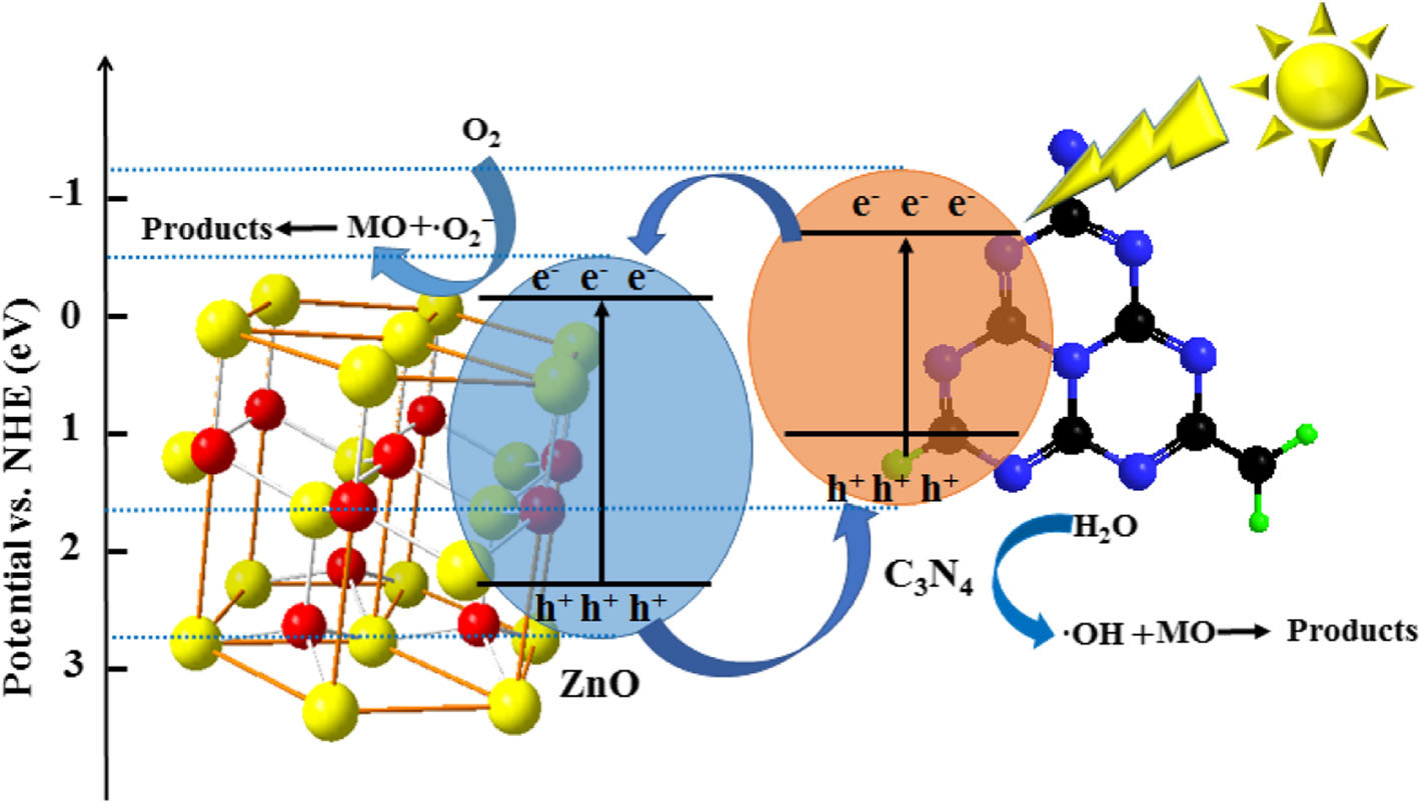
Mechanism for photocatalytic degradation of monazo dyes on the ZnO/Fe_3_O_4_/g-C_3_N_4_-50% photocatalyst

Based on the above discussion, it was concluded that the photocatalytic activity of the ZnO/Fe_3_O_4_/g-C_3_N_4_-50% nanocomposite semiconductor was significantly improved. This was because of the following two reasons: (1) heterostructure between g-C_3_N_4_ and ZnO improved the light absorption properties, and (2) the synergistic effect of the internal electric field and the matched band structure of g-C_3_N_4_ and ZnO increased the separation rate of photogenerated carriers (Fig. 14).

## Conclusions

In this study, ternary magnetic ZnO/Fe_3_O_4_/g-C_3_N_4_ nanocomposites were successfully fabricated, as novel recyclable visible-light-driven photocatalysts. Among all the prepared photocatalysts, the ZnO/Fe_3_O_4_/g-C_3_N_4_-50% composite photocatalyst exhibited the most efficient photocatalytic activity, due to the improved light absorption properties resulting from the heterojunction structure between g-C_3_N_4_ and ZnO, in addition, to the synergistic effect of their internal electric field and matched energy band structure. Moreover, the separation rate of the photogenerated carriers was high. The degradation efficiencies of MO, AYR, and OG over ZnO/Fe_3_O_4_/g-C_3_N_4_-50% were 97.87%, 98.05%, and 83.35%. This was due to the number of dye molecule adsorbed on the photocatalyst, and the structure of the azo dye molecule had an influence on the degradation. The kinetics of the degradation of MO on the composite photocatalyst was in accordance with first-order kinetics. Furthermore, the addition of Fe_3_O_4_ significantly improved the stability and recyclability of the photocatalyst. Superoxide ions are the main reactive species, which indicates that the azo dyes have the same degradation mechanism.

## Abbreviations

AO: Ammonium oxalate
AYR: Alizarin yellow R
CB: Conduction band
DRS: Diffuse reflectance spectroscopy
EDS: Energy dispersive X-ray spectroscopy
EIS: Electrochemical impedance spectroscopy
EtOH: Ethanol
Fe_3_O_4_: Ferroferric oxide
g-C_3_N_4_: Graphite-like carbon nitride
MO: Methyl orange
OG: Orange G
*p*-BQ: p-Benzoquinone
PL: Photoluminescence spectra
*t*-BuOH: Tert-butanol
TEM: Transmission electron microscopye
UV: Ultraviolet
VB: Valence band
VSM: Vibrating sample magnetometry
XRD: X-ray diffraction
ZnO: Zinc oxide
